# First αvβ6-integrin targeted radioligand therapy of breast cancer using Lu-177-Therahexin

**DOI:** 10.1007/s00259-026-07826-1

**Published:** 2026-02-26

**Authors:** Michael C. Kreissl, Jan Wuestemann, Elisabeth Eppard, Daniel Hescheler, Joanna Wybranska, Dennis Kupitz, Falco Reissig, Frankis G. Almaguel, Johannes Notni

**Affiliations:** 1 Division of Nuclear Medicine, Department of Radiology and Nuclear Medicine, University Hospital Magdeburg, Leipziger Strasse 44, Magdeburg, 39120 Germany; 2 TRIMT GmbH, Carl-Eschebach-Str. 7, Radeberg, 01454 Germany; 3 Center for Health Disparities and Molecular Medicine, Department of Basic Sciences, Loma Linda University School of Medicine, Loma Linda, CA 92350 USA; 4 Cancer Center, Loma Linda University Health, Loma Linda, CA 92354 USA; 5 Institute of Pathology, School of Medicine and Health, Technical University of Munich, Trogerstr. 18, Munich, 81675 Germany

**Keywords:** Breast Cancer, αvβ6-Integrin, Theranostics, Single-photon emission computed tomography, Planar scintigraphy

Breast cancer (BC) is the most common cancer in women. The “cancer integrin” αvβ6 is highly expressed on various carcinoma cell types, including BC [1, 2]. Feasibility of PET/CT imaging of BC using αvβ6-integrin targeted probes has been demonstrated previously [3], but corresponding radioligand therapeutics, e.g., labeled with ^177^Lu, have not been explored in this context.

We herein report the first αvβ6-integrin targeted radioligand therapy (RLT) of BC with ^177^Lu-Therahexin-503 [4] for a female patient (56 y, 64 kg) with metastatic breast cancer (initially HER2 amplification; currently ER pos, PR neg, HER2 1+, FISH neg, Ki-67 up to 41%; genomics: GATA3 gain-of-function, CDKN2A/B copy number loss, HRD neg), diagnosed 31 m ago with BC following detection of lytic bone lesions, after multiple conventional therapy lines (Letrozol+Trastuzumab+Pertuzumab 30–25 m ago; Trastuzumab-Deruxtecan (Enhertu) and palliative RTx 25–7 m ago; switch to Vinorelbin + PHESGO after radiological progress; progressing since 4 m with a CEA rising from 63 to 191 ng/mL, currently denosumab and pain palliation). The image shows αvβ6-integrin targeted posttherapeutic SPECT/CT (3 d p.i.; **A**, ventral MIP; **B**, coronal fusion, **C**–**F**, axial fusion) and planar scintigraphy (**G**; 2 h, 2 d, and 3 d p.i.; a, anterior; p, posterior) following administration of ^177^Lu-Therahexin-503 (7.124 GBq). 500 mL Gelafusal^®^ (4% succinylated gelatin, *M*_W_ 27–33 kDa, formulated in Ringer’s acetate) was infused over 6 h to reduce kidney uptake [4, 5]. The application was well tolerated without adverse effects. High uptake of the radiopharmaceutical was observed in the tumor manifestations in the bone/bone marrow as well as liver and lymph node metastases. Red arrows indicate lesions selected for exemplary tumor dosimetry (➀: lymph node, 13.7 Gy; ➁, ➂, ➃: bone, 22.8, 21.8 and 45.8 Gy, respectively; ➄, liver, 5.7 Gy), resulting in an estimated median dose of 3.1 Gy/GBq [0.8–6.4] comparable to ^177^Lu vipivotide tetraxetan (Pluvicto^®^, 4.4 Gy/GBq) [6]. Mean kidney dose was 4.7 Gy (0.66 Gy/GBq), which is slightly more than for Pluvicto^®^ (0.43 Gy/GBq) [7]. SPECT/CT fusion images (**B**–**F**) demonstrate high and specific uptake in BC metastases and low background retention, further corroborated by planar scintigraphy (**G**). 6 weeks after RLT, CEA had stabilized at 167 ng/mL, prompting continuation with further cycles. In conclusion, the high and persistent tumor uptake of ^177^Lu-Therahexin and the high doses delivered to BC tumor lesions suggests a clinical potential of this agent for RLT of BC patients particularly in cases refractory to conventional and advanced therapy lines such as Enhertu^®^, which represents a substantial unmet medical need.


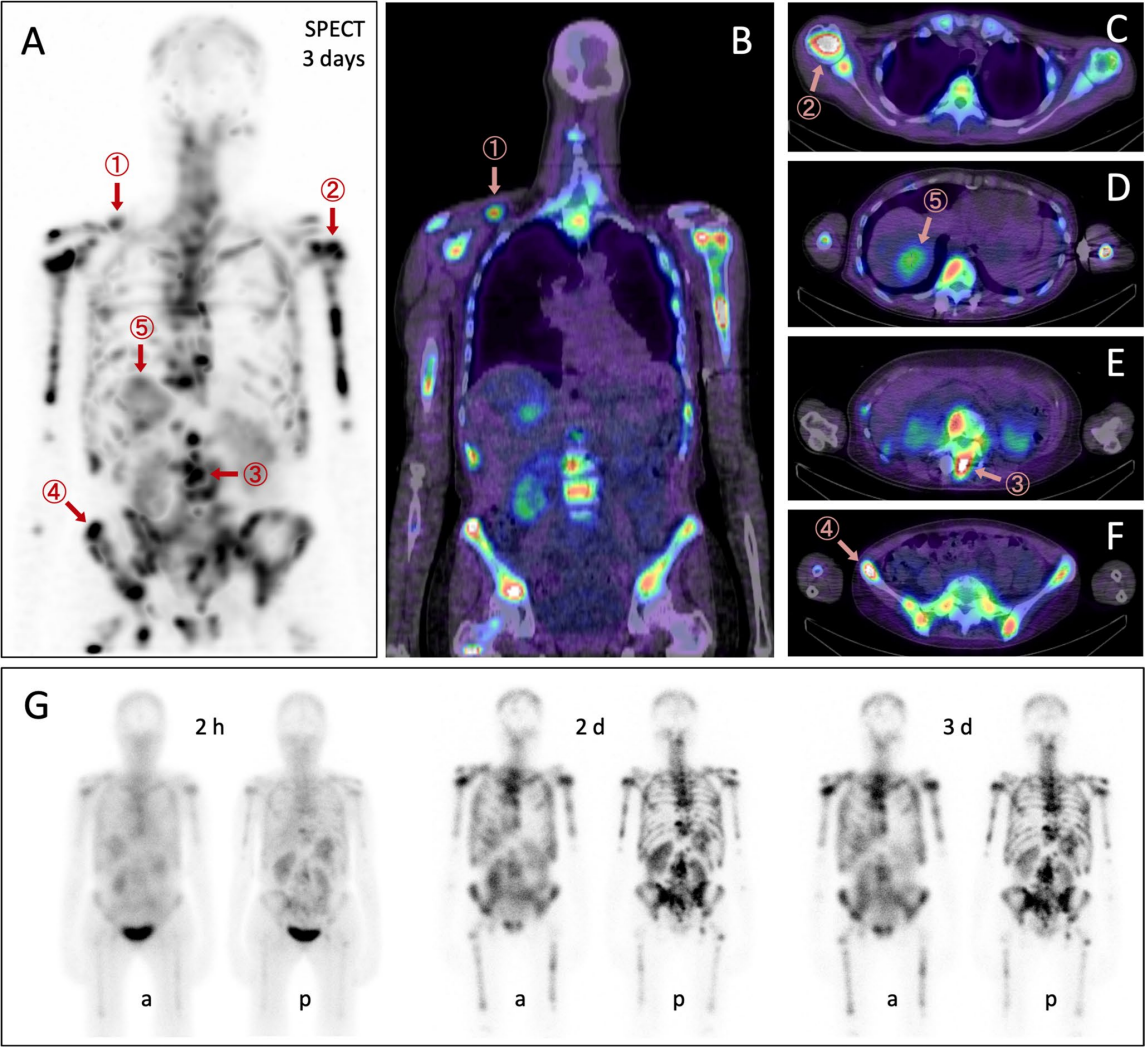


## Data Availability

The datasets used and/or analysed during the current study are available from the corresponding author on reasonable request.
